# The Influence of Loop Heat Pipe Evaporator Porous Structure Parameters and Charge on Its Effectiveness for Ethanol and Water as Working Fluids

**DOI:** 10.3390/ma14227029

**Published:** 2021-11-19

**Authors:** Krzysztof Blauciak, Pawel Szymanski, Dariusz Mikielewicz

**Affiliations:** Faculty of Mechanical Engineering and Shipbuilding, Gdansk University of Technology, Narutowicza 11/12, 80-233 Gdansk, Poland; k.blauciak@frigoconsulting.com (K.B.); dmikiele@pg.edu.pl (D.M.)

**Keywords:** Loop Heat Pipe, porous materials, mass transfer, heat transfer, phase transitions

## Abstract

This paper presents the results of experiments carried out on a specially designed experimental rig designed for the study of capillary pressure generated in the Loop Heat Pipe (LHP) evaporator. The commercially available porous structure made of sintered stainless steel constitutes the wick. Three different geometries of the porous wicks were tested, featuring the pore radius of 1, 3 and 7 µm. Ethanol and water as two different working fluids were tested at three different evaporator temperatures and three different installation charges. The paper firstly presents distributions of generated pressure in the LHP, indicating that the capillary pressure difference is generated in the porous structure. When installing with a wick that has a pore size of 1 μm and water as a working fluid, the pressure difference can reach up to 2.5 kPa at the installation charge of 65 mL. When installing with a wick that has a pore size of 1 μm and ethanol as a working fluid, the pressure difference can reach up to 2.1 kPa at the installation charge of 65 mL. The integral characteristics of the LHP were developed, namely, the mass flow rate vs. applied heat flux for both fluids. The results show that water offers larger pressure differences for developing the capillary pressure effect in the installation in comparison to ethanol. Additionally, this research presents the feasibility of manufacturing inexpensive LHPs with filter medium as a wick material and its influence on the LHP’s thermal performance.

## 1. Introduction

LHPs are very efficient heat transfer devices operating passively where the principle of operation is based on evaporation and condensation of the working fluid at a specific pressure related to the required conditions. In such a two-phase passive thermal control apparatus, extensive amounts of heat can be transferred with stable control of the heat source temperature. There has been a widespread effort to extend successful applications of LHPs to more common terrestrial applications [1,2,3,4,5,6,7,8] in order to develop more passive cooling systems, mainly based on liquid–vapour phase-change mechanisms to remove large heat fluxes. The electronic terrestrial applications benefit from the cooling advantages of LHPs (e.g., passive—electrical power-free, long-distance heat transfer, flexibility in design and assembly, robustness, antigravity capability, noise and vibration-free operation).

The demand for cooling and thermal management of electronic devices increases over the limits of the current state-of-the-art cooling technologies, which primarily result in challenges towards miniaturisation of electronics and transfer higher heat fluxes from the electronic components produced at the present day by the space and terrestrial electronics industry.

The wick structure installed in the evaporator is responsible for providing a high capillary pressure to circulate the working fluid in the system. The most important parameters that characterise the wicks are permeability, thermal conductivity, capillary pumping performance, effective pore radius, interface heat transfer and wettability [9,10,11]. These parameters are determined by the internal wick structure and material properties and depend on the manufacturing process of the wick itself. According to the literature [9,12], most porous structures used in LHPs have been made of metallic materials, such as nickel, titanium, aluminium, stainless steel and, occasionally, ceramic, polymer and either silicone or foam. The most widespread technology for the manufacturing of metal wicks is sintering.

Nowadays, several laboratories endeavour to find a novel method of fabrication of wick or new materials which provide high capillary forces and high permeability or mass flow rate (e.g., additive manufacturing (AM)—colloquially known as 3D printing) [9,10,11,13,14,15], as these two design features are typically inhibitive of each other. AM is a very promising method of wick or LHP manufacturing; however, it is still costly and needs a lot of research to be conducted in this area. The main drawback is currently the minimum pore size that can be manufactured using AM, which limits the use of AM LHPs in long-distance transport applications.

Some scholars make attempts to manufacture a low-cost, functional LHP [16] or utilise the porous material manufactured by filter appliances companies as an LHP wick (e.g., Siedel [17]). Some attempt to use commercially available porous structures such as, for example, stainless steel sintered porous structures. Hence, this research is a continuation of the work carried on by Mikielewicz et al. [18,19,20] and presents the feasibility of manufacturing economical LHPs with sintered stainless steel powder by Tridelta Siperm GmbH (Dortmund, Germany) [21] as a wick material and its influence on LHPs thermal performance.

This paper presents studies of the capillary effect in commercially available stainless steel porous structures with different pore sizes of which the wick of the LHP evaporator is made. Two working fluids were considered in the tests, namely water and ethanol, at three different evaporation temperatures. Different installation charges were considered and compared to find an appropriate amount of working fluid inventory and its influence on LHP thermal performance. Characteristics of the distributions of pressure increase and mass flow rate in the function of heat flux were presented and discussed.

## 2. Experimental Rig

**Figure 1:**
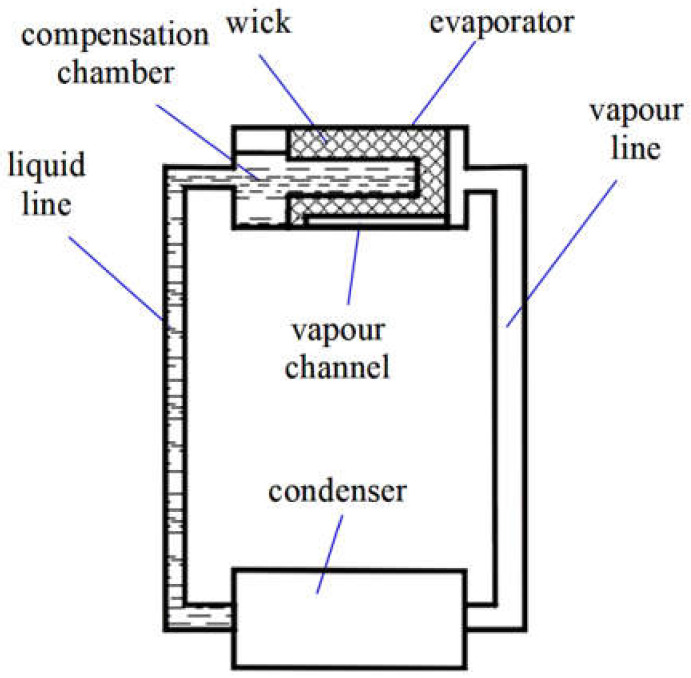
The layout of LHP [20].

The LHP evaporator with the sintered stainless steel porous wick was manufactured, assembled and tested to evaluate the possible capillary pressure difference created by the porous structure within the evaporator and its thermal performance. The evaporator was designed to enable the wick to be exchanged to a different one with another pore size. The experiment consisted of measuring the pressure rise in the evaporator while changing the applied heat load to the evaporator casing, resulting in different thermal and operational conditions. The test facility is schematically presented in Figure 1.

The principle of LHP operation is rather straightforward [1,2,20]. When heat is supplied to the evaporator, the meniscus is formed at the liquid/vapor interface in the evaporator wick, generating the required capillary forces to pump the fluid. Surface tension developed in a wick is a source of the pumping force used to circulate the fluid in the loop. The produced vapor flows down through the system of grooves then to the evaporator, where the capillary pressure pushes out the vapor in the direction of the vapor line towards the condenser rendering the fluid transport around the loop. The compensation chamber (CC) serves for storing and sustaining the surplus of working fluid and control of LHP operation.

**Figure 2:**
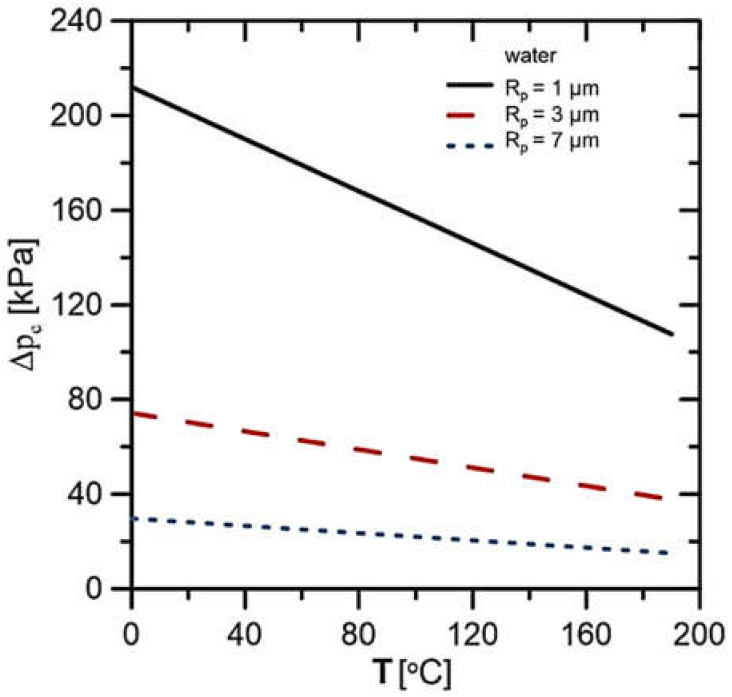
Capillary pressure rise characteristic for water as a working fluid.

**Figure 3:**
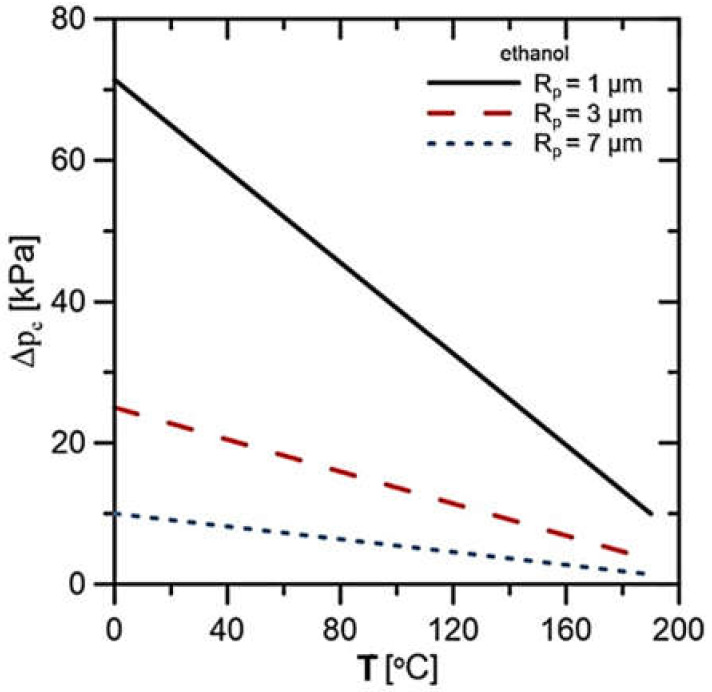
Capillary pressure rise characteristic for ethanol as a working fluid.

Considering the large variety of working fluids possible to apply in the LHP installation, it was decided to use the most common and previously applied working fluids in LHPs. Based on this analysis, the rig design requirements, physical properties of the working fluid and its possibility of generating the largest capillary pressures for further experiments were selected two fluids, namely distilled water and technical-grade ethanol. Such fluids are suitable for analysis due to their high potential to generate a capillary pressure difference in the porous structure (∆p_c_). A capillary pressure difference (∆p_c_ = 2σ/R_p_) depends on the surface tension of the working fluid (σ) and pore radius (R_p_). Characteristics of the theoretically feasible capillary pressure rise as a function of temperature for water are presented in Figure 2 and for ethanol in Figure 3. Both cases presented the effect of pore size on the generated pressure rise. In the considered temperature range, it can be seen that water has a higher potential to create a capillary pressure rise for each of the considered wick pore radiuses. The highest values of ∆p_c_ are obtained for the smallest values of the pore radius. With increasing temperature, the potential to generate ∆p_c_ decreases.

Table 1 presents the basic physical properties of the selected working fluids. The data were determined using the REFPOROP 10.0 software [22]. In the installation filled with water, triple distilled water was used to avoid undesirable corrosion effects or the formation of sediments inside the LHP elements during the evaporation and condensation processes, while in the case of the second working fluid, technical-grade ethanol with a concentration of 99.7% was used.

In order to illustrate the changes in individual physicochemical parameters and dimensional numbers as a function of temperature, an extract of the values of selected physical properties of fluids for several selected operating temperatures of the LHP is presented (Table 2). The data were determined using the REFPROP 10.0 software.

**Figure 4:**
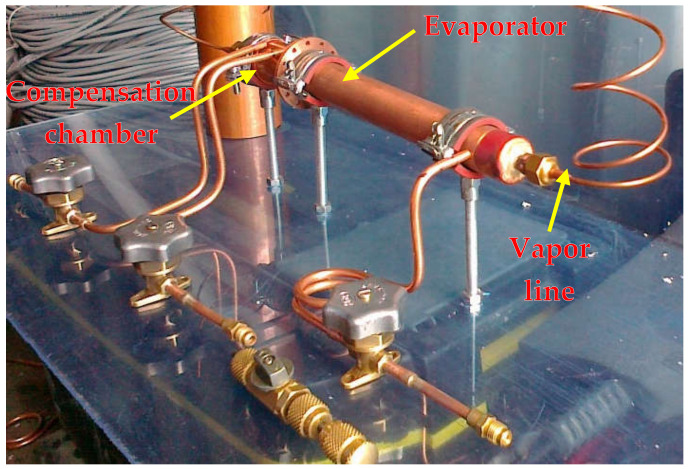
Photograph of the evaporator.

**Figure 5:**
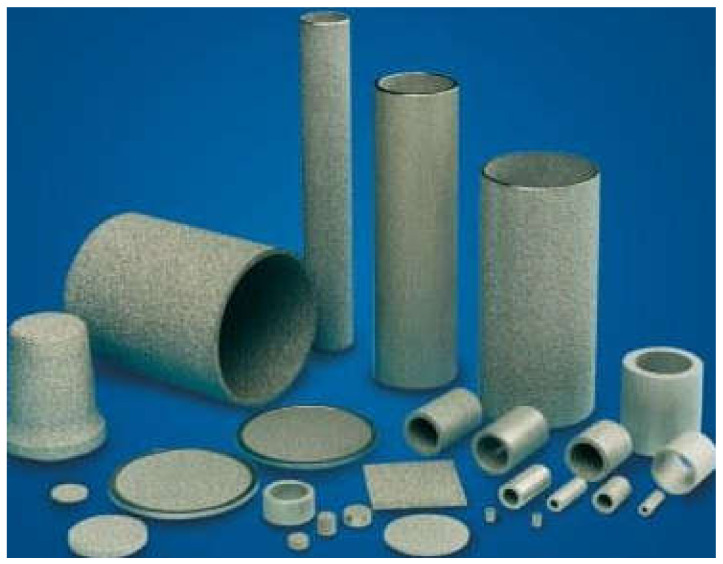
Examples of the wick manufactured by Tridelta Siperm GmbH [20].

**Figure 6:**
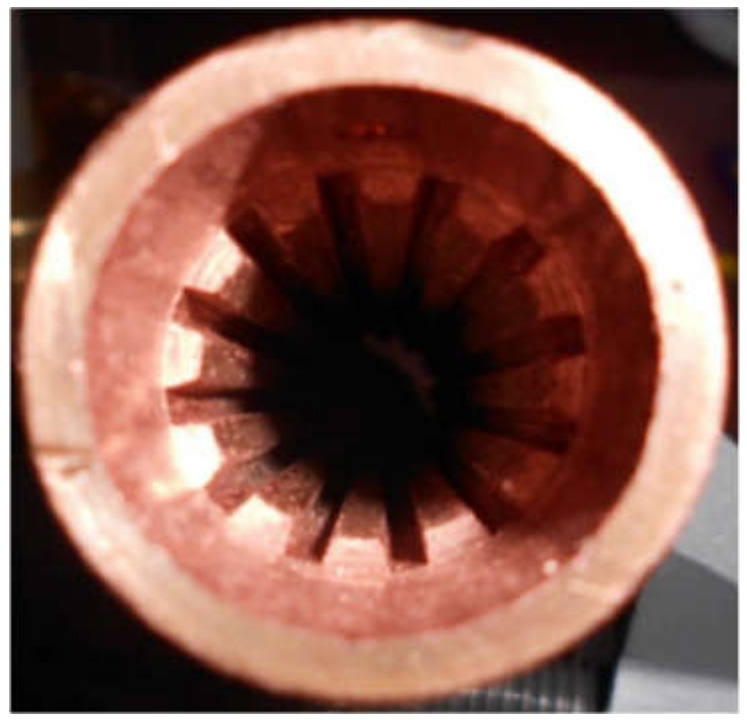
Cross-section of evaporator casing [20].

**Figure 7:**
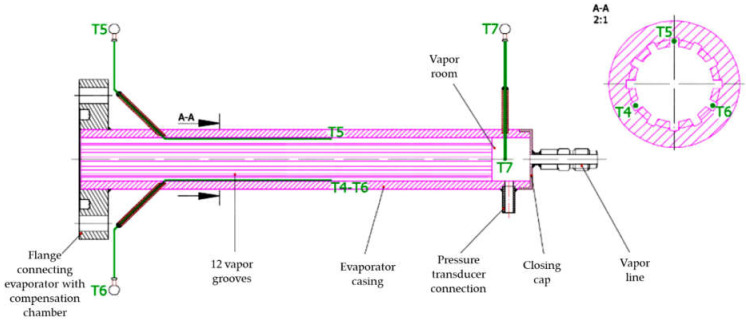
View of the evaporator with connections to thermocouples and pressure transducer.

In this study, the evaporator filled with three different wick materials was tested. The evaporator design allows for the exchange of the porous wick. The outline of the evaporator is presented in Figure 4. A sintered stainless steel cylindrical wick material was manufactured by Tridelta Siperm GmbH, a provider of porous metals, and inserted inside the evaporator casing (Figure 5) [20]. The porous wicks have a mean pore radius of 1 μm, 3 μm and 7 μm, and porosity of 24%, 33% and 35%, respectively. Such material was selected for its high resistance to corrosion and chemical compatibility with water and ethanol [23]. The evaporator casing was made of copper. On the internal side of the evaporator’s casing, 12 longitudinal vapor grooves necessary for transporting vapor to the evaporator outlet were drilled. The cross-section of the evaporator casing is presented in the photo (Figure 6) and the schematic (Figure 7). The entire length of the evaporator is 216.5 mm.

One of the most difficult to design and the most important elements of LHP is the CC, as it is responsible for the control of pressure and temperature in the system as well as hydrodynamics within the loop. Following several tests, the volume of the CC was set to 0.043 dm^3^ for the assumed dimensions of the evaporator. The sealed flange connection combined the evaporator and the CC, guaranteeing tightness and the possibility to exchange the wick and perspective evaporator revisions. Inside the CC, two thermocouples were installed to observe the temperature gradient inside during the application of LHP under different thermal loads.

The thermal load was applied using the electrical resistance wire. The wire was wound around the evaporator casing and connected to the laboratory DC supplier with adjustable current and voltage. Knowledge of the latter enabled calculation of the effective electric power applied to the resistor. Assuming that there was no heat loss through the insulation in the heating zone, the applied electrical power was taken as the rate of heat supplied to the system. Transport lines were made of smooth-wall copper tubes. The length of the liquid line length was 1152 mm (including bayonet) with an internal diameter equal to 2 mm. The vapor line length was 880 mm with an internal diameter of 2.95 mm. Condenser cooling was obtained using water circulating in a closed loop. Circulation of water was provided using a circulation pump featuring a flow rate up to 0.175 L/min. In parallel, the experimental rig was equipped with a visualisation section made from a transparent glass tube enabling the inspection of working fluid flow structures. Therefore, a two-way valve was installed to enable direct working fluid flow either through the copper vapor line or the transparent vapor line.

**Figure 8:**
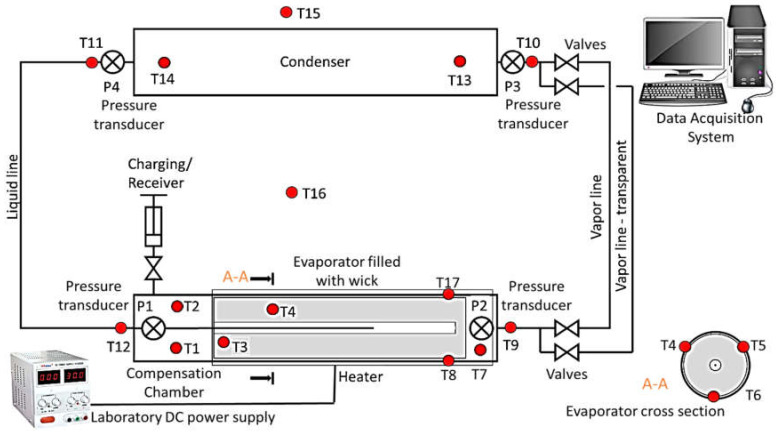
General schematic of LHP experimental rig with an indication of the measurement points.

**Figure 9:**
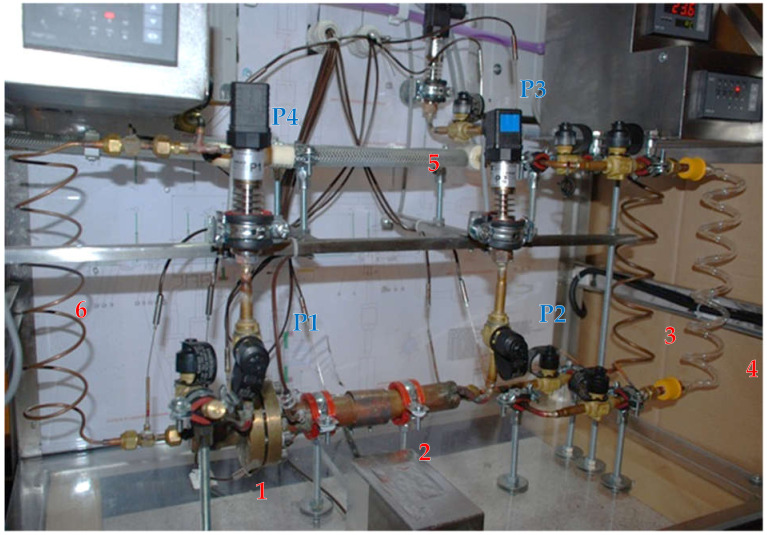
Photograph of the test facility with marked measurement points (before insulation): 1—compensation chamber; 2—evaporator; 3—vapour line; 4—glass tube for inspection of flow structure in vapour line; 5—condenser; 6—liquid line; P1, P2, P3, P4—pressure transducers.

Figure 8 presents the outline of the LHP experimental facility where the location of all measuring points is indicated, whereas Figure 9 shows is the general view of the rig.

The following measurement instrumentation was performed using:

1. Eighteen class 1, T-type thermocouples with measurement error equal to ±(2.0 × 10^−3^ × [T] + 0.3 °C + number) integrated with multiplexer EMT200. Locations of thermocouples are shown in Figure 7 and Figure 8;
2. Four class 0.1 pressure transducers with the measurement range of 1000 kPa of absolute pressure. Locations of pressure transducer measurement points are presented in Figure 8 and Figure 9;
3. Digital multimeter UT71E for the recording of electrical power supplied to the evaporator through the resistance wire (measurement accuracy ±(2% + 50)).

Before measurements, the test rig was insulated using mineral wool with an aluminium coating to reduce the heat dissipation from the evaporator test section. In addition, the liquid and vapor lines were insulated using synthetic rubber of 13 mm thickness.

Each of the measurements was proceeded by installation vacuuming using a two-stage vacuum pump (model CPS VP6D). The maximum possible vacuum level of 99.999% (1.95 × 10^−3^ kPa abs) was normally achieved. Before the LHP startup, the condenser cooling section was filled with water. All measuring devices were connected and linked to the camera recorder. Then, using a special applicator, the installation was charged with working fluid. In order to obtain reproducible condensation conditions, the condenser chiller was initiated before the heating section until the desired steady temperature was obtained. The next steps consisted of a setup of the heating section recorder, launching the automatic temperature transducers, time and heating section recorders (with an automatic measurement recorder at 1 s intervals) and a recorder of pressure values displayed on panel displays (with a camera recording at 60 s intervals). The average measurement duration was determined to be about 120–130 min. Each of the measurement series was carried out for three evaporator temperature settings with the same working fluid volume. Therefore, each subsequent measurement required the use of cooling water in the thermostat with a similar temperature level. After the measurement with the last third heater setting, the installation was emptied from the working fluid, and then the above-mentioned procedure was repeated from the beginning by changing the parameters according to the previously described configuration of the test procedure.

The measurement uncertainties were estimated based on the analysis of systematic component errors of the measurement system [24] and presented in Table 3.

## 3. Results

As mentioned earlier, the experimental analysis consisted of experiments at three different working fluid charges, 60 mL, 65 mL and 70 mL, three different wicks featuring pores of 1 μm, 3 μm and 7 μm, and three different evaporator casing temperatures, T_w_ = 90 °C, T_w_ = 100 °C and T_w_ = 110 °C. Two different working fluids were tested: water and ethanol. In total, 12 saturated pressure levels were recorded in the case of water and 18 saturation pressure levels in the case of ethanol, while the thermal load was varied. This paper presents the results of pressure difference possible to reach in the evaporator with a porous wick for two installation charging ratios and two different fluids. The range of investigated parameters is presented in Table 4. The subsequent discussion is given for the case of a single filling volume of 65 mL and two test fluids.

**Figure 10:**
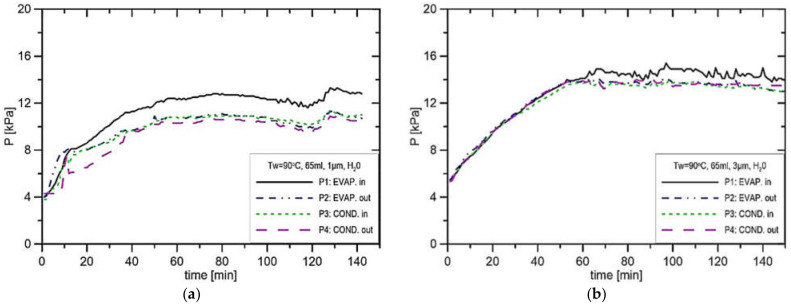
Distribution of pressure in function of time in case of water, charge volume 65 mL, T_w_ = 90 °C, (**a**) R_p_ = 1 μm, (**b**) R_p_ = 3 μm.

**Figure 11:**
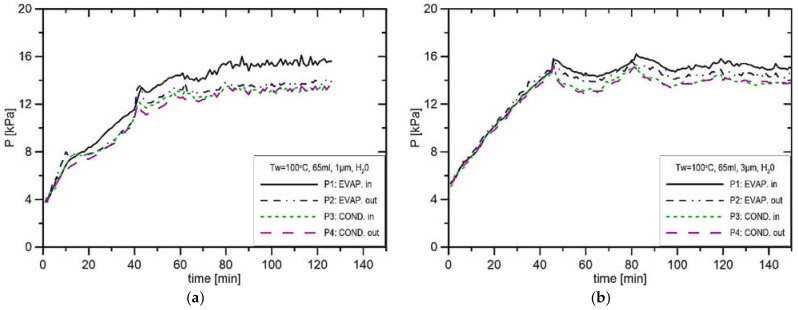
Distribution of pressure in function of time in case of water, charge volume 65 mL, T_w_ = 100 °C, (**a**) R_p_ = 1 μm, (**b**) R_p_ = 3 μm.

**Figure 12:**
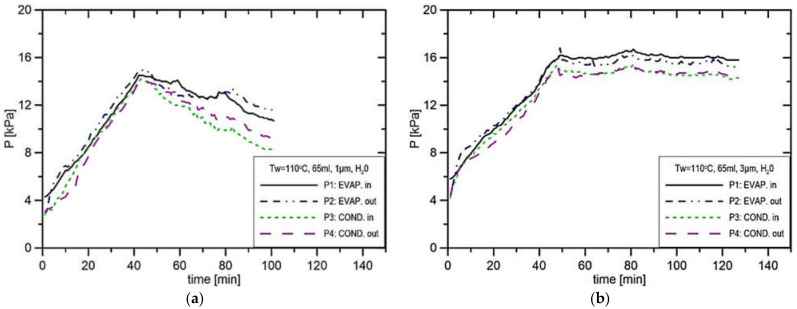
Distribution of pressure in function of time in case of water, charge volume 65 mL, T_w_ = 110 °C, (**a**) R_p_ = 1 μm, **(b**) R_p_ = 3 μm.

**Figure 13:**
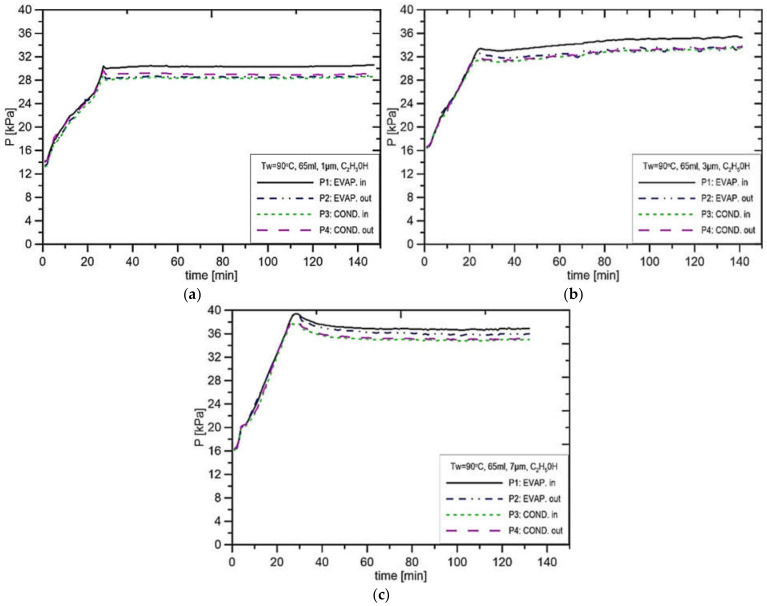
Distribution of pressure in function of time when using ethanol, charge volume 65 mL, T_w_ = 90 °C, (**a**) R = 1 μm, (**b**) R_p_ = 3 μm, (**c**) R_p_ = 7 μm.

In the case of water, the results of pressure distributions are presented in Figure 10, Figure 11, Figure 12 and Figure 13 for the charge volume of 65 mL. Analysis of the developed pressure in the case of water as a working fluid shows the pressure values before and after the evaporator, which vary with respect to the applied evaporator saturation temperature and the pore size. The level of pressure drop in the vapor and liquid lines is much smaller with respect to the pressure difference P2–P1, where P2 is the pressure after the evaporator (the highest pressure in the loop). P1 is the pressure before CC (the lowest pressure in the loop). Some pressure fluctuations are observed in all pressure distributions; however, consistent pressure differences are noticed in the distributions. During the investigations, the measurement run lasted for about 2.5 h, from which we can detect that reaching the steady-state conditions lasted for the first 30 min.

In Figure 10a, the rate of pressure increase is about 0.44 kPa/s, and the maximum pressure difference P1–P2 was equal to 2.55 kPa. In the experiment presented in Figure 10b, the pressure P1 stabilises at the level of 14.1 kPa, while in the vapor line, it stabilises at 12.9 kPa. The only difference between the experiment presented in Figure 10a,b is the size of the pore, which was changed from 1 μm to 3 μm. That confirms the fact that the reduction in the pore size leads to an increase in produced pressure. Figure 10a and Figure 11a presented a similar situation to those presented in Figure 10b and Figure 11b; however, the evaporator casing temperature increased from 90 °C to 100 °C. In this case, the pressure difference in the installation P1–P2 amounts merely to 2.2 kPa. In the experiment presented in Figure 11a, the pressure in the CC settled at the level of 15.0 kPa and 13.6 kPa in the vapor line, which results in the maximum pressure difference in the installation of 1.4 kPa. This suggests that the increase in evaporator casing temperature from 90 °C to 100 °C reduces the potential to produce the capillary pressure difference. In the experiments presented in Figure 12a,b, the evaporator casing temperature was set to 110 °C, whereas the pore size was equal to 1 μm and 3 μm, respectively. In the case of the run presented in Figure 12b, the pressure in the vapor line was 14.2 kPa, and the pressure in the CC was 15.9 kPa, which results in the maximum pressure difference in the installation equal to 1.7 kPa.

**Figure 14:**
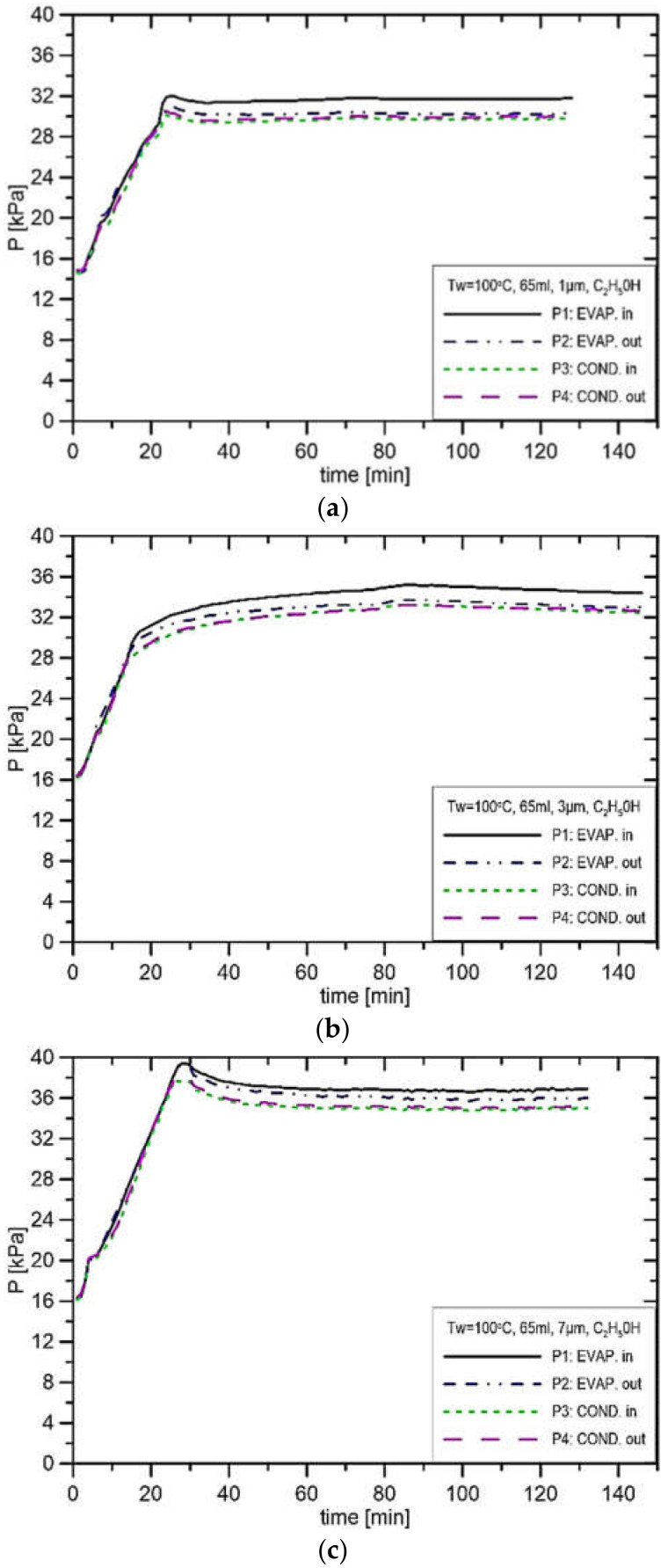
Distribution of pressure in function of time when using ethanol, charge volume 65 mL, T_w_ = 100 °C, (**a**) R_p_ = 1 μm, (**b**) R_p_ = 3 μm, (**c**) R_p_ = 7 μm.

**Figure 15:**
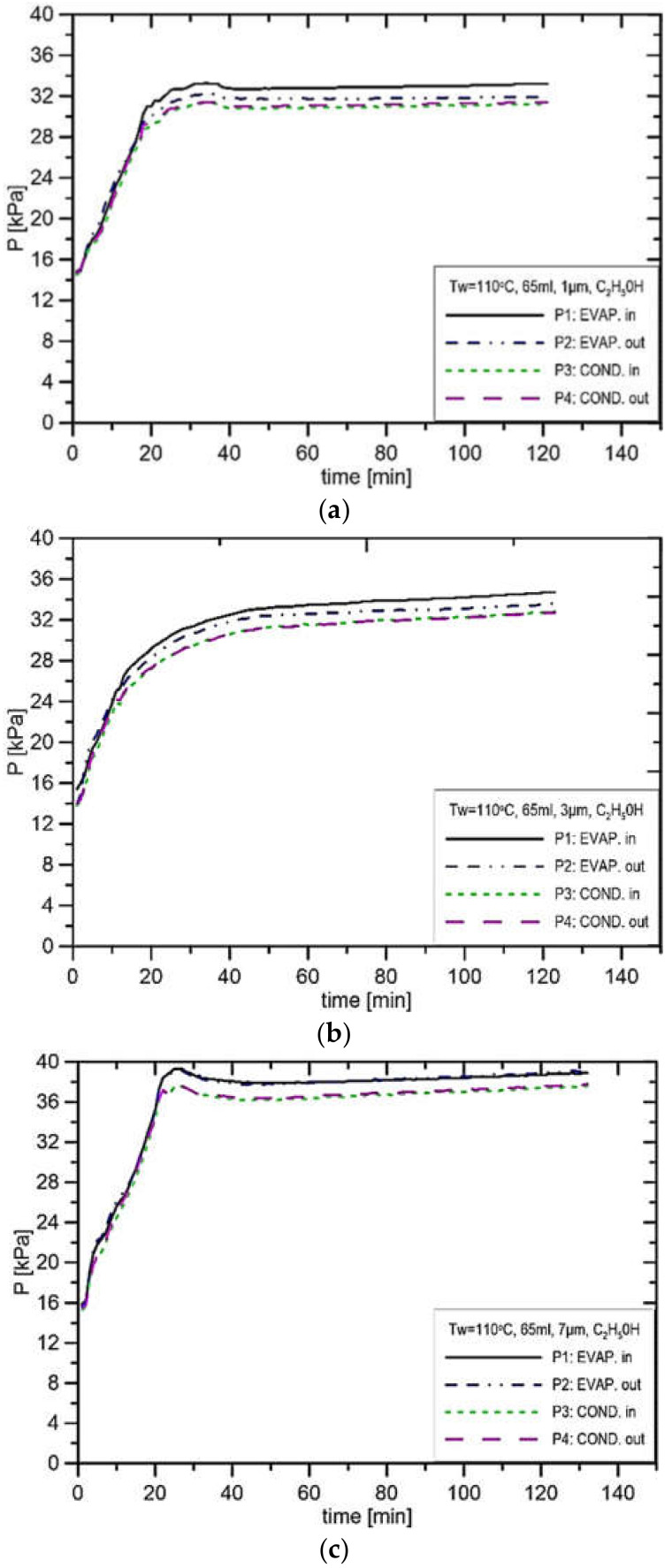
Distribution of pressure in function of time when using ethanol, charge volume 65 mL, T_w_ = 110 °C, (**a**) R_p_ = 1 μm, (**b**) R_p_ = 3 μm, (**c**) R_p_ = 7 μm.

The analysis of the pressure distribution for ethanol as a working fluid and filling volume of 65 mL was presented in Figure 13, Figure 14 and Figure 15. The general observation is that the pressure drops in the vapor and liquid lines are smaller in comparison to the pressure difference in the evaporator. Another observation is that the pressure fluctuations are generally smaller than in the case of water as a working fluid, although the operating pressure of the loop is much higher than in the case of water.

For experiments with ethanol as a working fluid, the experimental run lasted for 2.5 h, and 30 min was related to the startup. The difference from the case of water was much higher initial pressure in the installation.

In Figure 13a, the initial pressure stabilised at the level of about 13.5 kPa, and due to the heat supply, the maximum pressure reached 30.2 kPa. In the case of other pore sizes as well as other evaporator casing temperatures, the pressure reached values of 40.0 kPa. In the case of the parameters presented in Figure 13b, the pressure in the CC settled at the level of 30.2 kPa, while in the vapor line, it settled at the level of 29.0 kPa. Therefore, the wick produces a pressure increase of 1.2 kPa for the evaporator casing temperature equal to 90 °C and the pore size of 1 μm. In the case of the experimental run presented in Figure 13b, where the pore size is equal to 3 μm, the pressure before the CC settled at 35.1 kPa, and the pressure in the vapor channel settled at the level of 33.9 kPa. This confirms that the reduction in the pore radius leads to an increase in pressure. Figure 13c presents the results for the case when the wick pore size is 7 μm. In that case, the pressure before the CC stabilised at the level of 37.1 kPa, whereas the pressure in the vapor line was at the level of 35.6 kPa. This indicates that the size of the pores has practically no influence on the capillary pressure difference.

Figure 14 presents the results of experiments where the evaporator casing temperature is 100 °C at the filling volume of 65 mL. From the results in Figure 14a, the pressure in the CC is 32.0 kPa, and the pressure in the vapor line is 29.9 kPa; hence, the pressure difference at the installation is 2.1 kPa. In the case presented in Figure 14b, the pressure in the CC settled at the level of 35.0 kPa, and in the vapor line, it settled at the level of 33.0 kPa, which gives a pressure difference in the installation of 2.0 kPa. This pressure difference is smaller than in the case of the pressure difference from Figure 14a, which confirms the fact that with the increase in the pore size, the potential to produce capillary pressure difference decreases. In the case of pore size equal to 7 μm (Figure 14c), the pressure in the CC stabilised at the value of 37.0 kPa, and in the vapor line, it stabilised at the level of 35.0 kPa. In Figure 15 presented are the results of experimental runs with the evaporator casing temperature set to 110 °C and three different pore sizes of 1 μm, 3 μm and 7 μm. A similar character of changes as in the case of evaporator temperature settings of 90 °C and 100 °C is present. From the comparison of the three values of heater setting, with the increase in evaporator casing temperature, at the same value of the pore size, the potential to produce the capillary temperature difference decreases. Such a conclusion can be drawn by comparing Figure 13a, Figure 14a and Figure 15a, where the pore size is 1 μm, and the difference between these cases is only in the evaporator temperature setting. Other comparisons at the same value of the pore size are shown in Figure 13b, Figure 14b and Figure 15b for the pore size of 3 μm, and Figure 13c, Figure 14c and Figure 15c for the pore size of 7 μm. Due to the change in the setting of wall temperature, the resulting pressure difference is 0.3 kPa.

### Determination of Mass Flow Rate of Working Fluid in the LHP Evaporator

An essential parameter needed for the analysis of the pressure rise is the mass flow rate of the working fluid passing through the evaporator. Due to the lack of the possibility of direct measurement of the mass flow rate, an attempt was made to estimate it for the tested conditions. By knowing the heat flux supplied to the evaporator casing and the pressure and temperature range measured at the inlet and outlet of the evaporator, the mass flow rate of the working fluid ($\dot{m}$*_f_*) inside the LHP was determined based on the heat balance. The mass flow rate was determined from the ratio of the heat flux ($\dot{Q}$) supplied to the evaporator casing divided by the enthalpy (*h*) difference between the evaporator outlet and evaporator inlet. Enthalpy was determined using the data from the REFPROP 10.0 software:

(1) $${\dot{m}}_{f}=\frac{\dot{Q}}{h_{evap.out}-h_{evap.inl}}$$

The results of the calculations are presented in Table 5.

The data presented in the table indicate the correlation between the heat source temperature and pore size, which, in the case of water, causes an increase in the mass flow rate as the temperature of the heat source increases and increases when the radius of the pore increases. Ethanol seems to be the most optimal with the pore size equal to 7 μm.

**Figure 16:**
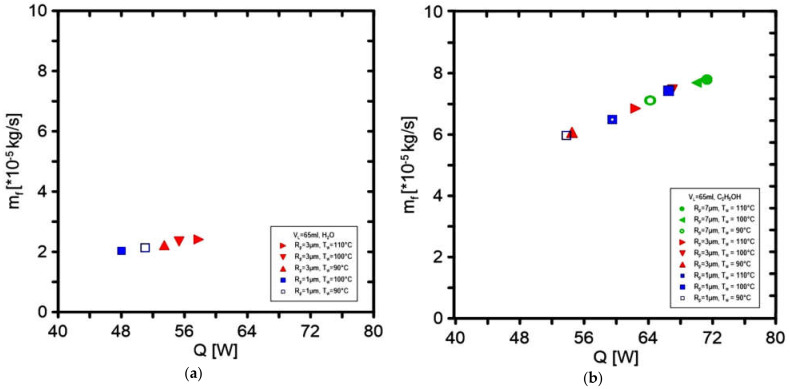
The mass flow rate in the function of applied heat flux for (**a**) water as a working fluid and (**b**) ethanol as a working fluid.

**Figure 17:**
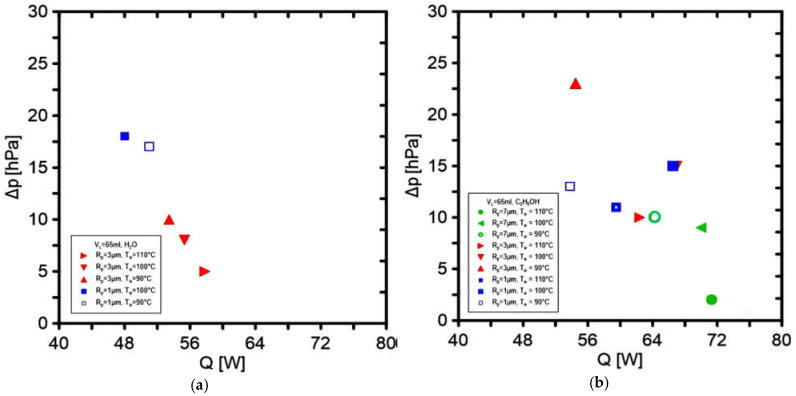
Pressure change in the evaporator in the function of applied heat flux for (**a**) water as a working fluid and (**b**) ethanol as a working fluid.

The use of ethanol in the experimental rig with the same operating parameters caused the observation of several times higher values of the mass flow rate regardless of the heat source setting, comparing the results with the use of distilled water. Figure 16 presents the dependence of the heat flux relation in the function of the achieved flow rate of the working fluid. The figures show that the relationship is practically linear. The influence of the pore radius on the vapor outlet and the applied temperature is noticeable. Figure 17 presents the dependence of the applied heat flux on the pressure increase where the eac measurement comes from a different experiment. For water, the experimental points are arranged in a linear trend, while in ethanol, the trend line was not linear, which means that the measurements were burdened with greater error.

## 4. Conclusions

The porous materials made of sintered stainless steel powder were tested as the evaporator wicks of the LHP. The experimental facility was assembled to study the efficiency of the various porous structures, characterised by the pore size of 1 μm, 3 μm and 7 μm for different working fluids. Two working fluids were tested, namely, water and ethanol.

The experiments indicate the crucial issue of adequate charge of installation with the working fluid. For both fluids considered, it was found that the pressure difference can reach up to 2.5 kPa for water as a working fluid and the pore size of 1 μm at the installation charge of 65 mL and 1.6 kPa in case the filling is 70 mL. This corresponds to 65% or 70% of the charge in installation. For other values of fillings, significantly lower values of pressure difference were obtained. In the case of ethanol, the results return a similar qualitative trend.

The respective values of mass flow rate were determined based on the energy balance for the evaporator and condenser separately. Good consistency of the results was obtained. The available mass flow rate is about 2.5 times higher in the case of ethanol than in the case of water at identical conditions.

Additionally, this research presents the feasibility of manufacturing inexpensive LHPs with filter medium as a wick material and its influence on the LHP’s thermal performance.

## Figures and Tables

**Table 1:** Comparison of the basic physical properties of water (H_2_O) and ethanol (C_2_H_5_OH).

Working Fluid	Critical Pressure	Critical Temperature	Molar Mass	Triple Point Temperature	Boiling Temperature
	[kPa]	[°C]	[kg/kmol]	[°C]	[°C]
H_2_O	22.064	373.95	18.015	0.01	99.974
C_2_H_5_OH	6268	241.56	46.068	−114.15	78.420

**Table 2:** Comparison of physicochemical parameters of water and ethanol versus temperature.

Working Fluid	Liquid Density	Vapor Density	Specific Heat of Liquid	Specific Heat of Vapor	Enthalpy of Vaporisation	Liquid Viscosity	Vapor Viscosity	Prandtl Number of Liquid	Prandtl Number of Vapor	Surface Tension
	[kg/m^3^]	[kg/m^3^]	[kJ/kgK]	[kJ/kgK]	[kJ/kg]	[μPas]	[μPas]			[N/m]
H_2_O|_T = 20_	998.16	0.017314	4.1844	1.9059	2453.5	1001.6	9.5441	7.0038	0.9979	0.072736
H_2_O|_T = 40_	992.18	0.051242	4.1796	1.9314	2406.0	652.72	10.185	4.3263	1.0037	0.069596
H_2_O|_T = 80_	971.77	0.293670	4.1969	2.0120	2308.0	354.04	11.539	2.2177	1.0089	0.062673
H_2_O|_T = 100_	958.35	0.598170	4.2157	2.0800	2256.4	281.58	12.232	1.7480	1.0138	0.058912
H_2_O|_T = 120_	943.11	1.122100	4.2435	2.1770	2202.1	232.03	12.927	1.4412	1.0245	0.054968
C_2_H_5_OH|_T = 20_	789.59	0.112410	2.5121	1.5840	926.61	1195.2	8.6186	18.054	0.7826	0.022414
C_2_H_5_OH|_T = 40_	772.47	0.321860	2.7565	1.6488	904.94	821.65	9.2312	14.009	0.8082	0.019886
C_2_H_5_OH|_T = 80_	734.64	1.759100	3.2036	1.8150	846.97	429.47	10.431	9.0044	0.8542	0.015030
C_2_H_5_OH|_T = 100_	713.14	3.530000	3.4048	1.9319	809.83	322.63	110.22	7.4024	0.8785	0.012713
C_2_H_5_OH|_T = 120_	689.39	6.568700	3.5983	2.0846	766.47	246.81	116.14	6.1681	0.9052	0.010481

**Table 3:** List of measurement errors.

	Error Designation
Temperature	±0.2 °C
Pressure	±25 Pa
Heat input	±1.3 W
Working fluid volume	±1 mL
Mass flow rate	±2 × 10^−5^ kg/s

**Table 4:** Range of investigated parameters.

Working Fluid	Charge Level [mL]	Heater Temp [°C]	Pore Size [µm]	P1–P2 [kPa]
WATER	65	90	1	2.5
		90	3	1.0
		100	1	2.2
		100	3	1.4
		110	1	0
		110	3	1.7
	70	90	1	1.5
		90	3	0.7
		100	1	1.6
		100	3	1.0
		110	1	0.9
		110	3	0
ETHANOL	65	90	1	1.2
		90	3	1.2
		90	7	1.5
		100	1	2.1
		100	3	2.0
		100	7	2.0
		110	1	1.9
		110	3	1.7
		110	7	0.3
	70	90	1	0.7
		90	3	1.1
		90	7	0.2
		100	1	0.8
		100	3	0.9
		100	7	0.2
		110	1	1.0
		110	3	0.8
		110	7	0.4

**Table 5:** Determination of the mass flow rate using the heat balance method.

Heater Temperature	Working Fluid	Pore Size	Heat Flux	Enthalpy-Evaporator Inlet	Enthalpy-Evaporator Outlet	Mass Flow Rate	P1–P2
[°C]	[-]	[μm]	[W]	[kJ/kg]	[kJ/kg]	[kg/s]	[kPa]
110	water	1	52.1	283.3	2642	2.08 × 10^−5^	1.6
100			48.5	278	2638	2.03 × 10^−5^	1.8
90			51.0	221.1	2621	2.13 × 10^−5^	1.7
110		3	57.8	243.7	2640	2.41 × 10^−5^	0.5
100			55.3	237.8	2608	2.33 × 10^−5^	0.8
90			53.4	231.5	2633	2.23 × 10^−5^	1.0
110	ethanol	1	59.5	337.6	1254	6.49 × 10^−5^	1.1
100			66.5	352.6	1247	7.4 × 10^−5^	1.5
90			53.8	331.3	1233	5.96 × 10^−5^	1.3
110		3	62.4	339.7	1249	6.86 × 10^−5^	1.0
100			67.0	342.6	1241	7.46 × 10^−5^	1.5
90			54.5	339.7	1236	6.08 × 10^−5^	2.3
110		7	71.3	359.2	1275	7.79 × 10^−5^	0.2
100			70.0	344.7	1255	7.69 × 10^−5^	0.9
90			64.2	342.3	1244	7.12 × 10^−5^	1.0
